# Genome-wide identification of thyroid hormone receptor targets in the remodeling intestine during *Xenopus tropicalis* metamorphosis

**DOI:** 10.1038/s41598-017-06679-x

**Published:** 2017-07-25

**Authors:** Liezhen Fu, Biswajit Das, Kazuo Matsuura, Kenta Fujimoto, Rachel A. Heimeier, Yun-Bo Shi

**Affiliations:** Section on Molecular Morphogenesis, Eunice Kennedy Shriver National Institute of Child Health and Human Development (NICHD), National Institutes of Health (NIH), Bethesda, Maryland 20892 USA

## Abstract

Thyroid hormone (T3) affects development and metabolism in vertebrates. We have been studying intestinal remodeling during T3-dependent *Xenopus* metamorphosis as a model for organ maturation and formation of adult organ-specific stem cells during vertebrate postembryonic development, a period characterized by high levels of plasma T3. T3 is believed to affect development by regulating target gene transcription through T3 receptors (TRs). While many T3 response genes have been identified in different animal species, few have been shown to be direct target genes *in vivo*, especially during development. Here we generated a set of genomic microarray chips covering about 8000 bp flanking the predicted transcription start sites in *Xenopus tropicalis* for genome wide identification of TR binding sites. By using the intestine of premetamorphic tadpoles treated with or without T3 and for chromatin immunoprecipitation assays with these chips, we determined the genome-wide binding of TR in the control and T3-treated tadpole intestine. We further validated TR binding *in vivo* and analyzed the regulation of selected genes. We thus identified 278 candidate direct TR target genes. We further provided evidence that these genes are regulated by T3 and likely involved in the T3-induced formation of adult intestinal stem cells during metamorphosis.

## Introduction

Thyroid hormone (T3) regulates the formation and/or maturation of many organs into the adult form during vertebrate development and also affects the homeostasis and physiological function of many adult organs/tissues, such as the heart and muscles^1–8^. T3-deficiency during development causes severe defects in mammals, including cretinism in human^3–5, 9, 10^. T3 exerts its effect on vertebrate development mainly during the so-called postembryonic development when high levels of T3 are present in the plasma, a period around birth in mammals and spanning from a few months prior to birth to several months after birth in human^3, 11^. How T3 affects mammalian development has been difficult to study in part because of the difficulty to manipulate the uterus-enclosed mammalian embryos. In addition, it has been shown that maternal T3 can also influence embryonic development in mammals^12–14^, complicating the analysis of T3 action *in vivo*.

Anuran metamorphosis resembles the postembryonic development in mammals and is totally dependent on T3^4, 15^. It can be easily manipulated by simply adding physiological levels of exogenous T3 to tadpole rearing water or blocking the synthesis of endogenous T3 in tadpoles. In addition, recent progress in genomic editing has also made it possible to analyze the function of endogenous genes in *Xenopus* ^16–20^. These and other properties have made anuran metamorphosis an excellent model to study the molecular basis of T3 action during vertebrate development. Studies on amphibian metamorphosis, especially in *Xenopus laevis* and *tropicalis*, have shown that T3 controls metamorphosis by regulating gene transcription through T3 receptors (TRs)^8, 21–37^.

Numerous studies have been carried out to identify genes regulated by T3 during metamorphosis. Early subtractive hybridization screening and subsequent gene expression microarray analyses have discovered many such genes in different organs/tissues during both natural and T3-induced metamorphosis^4, 21, 38–45^. These studies have revealed complex but informative global gene regulation patterns underlying organ transformations during metamorphosis. However, most of the genes thus identified are likely affected indirectly by T3, while only a few direct TR target genes have been characterized through traditional promoter analyses^46–50^. Here we have made use of the genomic sequence information available in *Xenopus tropicalis* (genome release version 4.1) to generate a tiled genomic probe set (479459 probes) of two microarray slides covering the sequences from 5.5 kb upstream to 2.5 kb downstream of the predicted transcriptional start sites of 17,355 *Xenopus tropicalis* genes (based on Ensembl release 46). This resulted in a probe-set that has about 40 probes (60-mer) tiled for each of the putative promoter regions at an average tiling distance of 205 bp. By using this genomic chip set and an antibody against *Xenopus* TRs, we then carried out a genome-wide chromatin immunoprecipitation (ChIP) assay on the intestine of premetamorphic tadpoles treated with or without T3. Nearly 300 genes were found to be bound by TR *in vivo* and more importantly, most were indeed found to be regulated by T3. Furthermore, bioinformatics analyses suggest that these genes are regulated during T3-dependent intestinal remodeling, implicating a role in the formation of adult intestinal stem cells during *Xenopus* metamorphosis, a period equivalent to the postembryonic development in mammals^3, 4^.

## Materials and Methods

### Experimental animals

Wild-type tadpoles of *Xenopus tropicalis* were obtained from NASCO, and developmental stages were determined according to Nieuwkoop and Faber^51^. Stage 54 tadpoles were treated for 2 days at 22 °C with 10 nM T3, close to the peak levels of T3 at the climax of metamorphosis in *Xenopus laevis* ^52^. All animal care and treatment were done as approved by Animal Use and Care Committee of Eunice Kennedy Shriver National Institute of Child Health and Human Development (NICHD), U.S. National Institutes of Health (NIH). The methods were carried out in accordance with relevant guidelines for the use of Xenopus tropicalis as a vertebrate model.

### Generation of luciferase reporter constructs for transcription assay

The firefly luciferase reporter constructs containing *Xenopus tropicalis* Dot1L (Dot1-Like) promoter (pTRE(Dot1L)-luc or *Xenopus tropicalis* Dot1L promoter with a mutant TRE (pmTRE(Dot1L)-luc were made based on pGL4.10 firefly luciferase vector (Promega) as previously described^53, 54^. The promoter constructs containing a putative TRE of selective genes (Table 1) were generated through PCR-mediated mutagenesis on the pmTRE(Dot1L)-luc as described^55^. In brief, each TRE-luc construct was made with primer set 1: 5′-TGTTGGATGCTCATACTCGTCC-3′ (LZ631) and TRE_R for a putative TRE (Table 1), and primer set 2: 5′-TRE_F for the same TRE (Table 1) and 5′-GGTAATGTCCACCTCGATATGTGC-3′ (LZ632) in two different PCR reactions to produce about 1 kb and 500 bp fragments, respectively. The two PCR fragments were gel purified and then mixed together as the templates for a second round of PCR with primers LZ631 and LZ632 to produce a ~1.5 kb fragment. The 1.5 kb fragment was then subjected to restriction enzyme digestion with Kpn I and Hind III and gel-purified. The 1048 bp restricted fragment was then subcloned into Kpn I-Hind III digested firefly luciferase reporter construct pmTRE(Dot1L)-luc, whereby the Dot1L mTRE in pmTRE(Dot1L)-luc was replaced with the TRE of the respective gene (Table 1). All the firefly luciferase reporter constructs were confirmed by sequencing.

**Table 1:** Putative TREs and primers for generating the firefly luciferase reporters.

Gene	Putative TRE	Primers (TRE sequences in bold)
MBD3	**GGGTCA**GATG**GGGACA**	TRE-F: 5′-CTAAG**GGGTCAGATGGGGACA**CCCGCGGGATTATTTATTTTATTC-3′ TRE-R: 5′-GCGGG**TGTCCCCATCTGACCC**CTTAGCCTGAAGTCTGAGG-3′
PPM1B	**AGGTCA**TTTG**AGGCCG**	TRE-F: 5′-CTAAG**AGGTCATTTGAGGCCG**CCCGCGGGATTATTTATTTTATTC-3′ TRE-R: 5′-GCGGG**CGGCCTCAAATGACCT**CTTAGCCTGAAGTCTGAGG-3′
PGPEP1	**GGTGCA**TGTC**AGGACA**	TRE-F: 5′-CTAAG**GGTGCATGTCAGGACA**CCCGCGGGATTATTTATTTTATTC-3′ TRE-R: 5′-GCGGG**TGTCCTGACATGCACC**CTTAGCCTGAAGTCTGAGG-3′
JUNB	**GGGTAA**TGTA**GGGTCA**	TRE-F: 5′-CTAAG**GGGTAATGTAGGGTCA**CCCGCGGGATTATTTATTTTATTC-3′ TRE-R: 5′-GCGGG**TGACCCTACATTACCCC**TTAGCCTGAAGTCTGAGG-3′
BEND7	**AGTTCA**GGGC**AGGTCA**	TRE-F: 5′-CTAAG**AGTTCAGGGCAGGTCA**CCCGCGGGATTATTTATTTTATTC-3′ TRE-R: 5′-GCGGG**TGACCTGCCCTGAACT**CTTAGCCTGAAGTCTGAGG-3′

### qRT-PCR

Total RNA was isolated from the intestine of tadpoles at premetamorphic stage 54, treated with or without 10 nM T3 at 22 °C for 2 days. The cDNA was prepared from 2.5 μg of total RNA using the Applied Biosystems’ High Capacity cDNA Archive kit according to the manufacturer’s instructions in a total volume of 50 μl. qRT-PCR based on SYBR Green detection was carried out to quantify gene expression levels on an ABI 7000 (Applied Biosciences) and *EF1*α (elongation factor 1α) was used as the normalization control as described previously^56^. The primers used for SYBR Green PCR were forward 5′-TCAAGCAACCAGTGACCAAG-3′ and reverse 5′-TTTCCCAGAAGAGCTGCCT-3′ for MBD3, forward 5′-GATGTCATGAGCAACGAGGA-3′ and reverse 5′-TCACGGCTTCCCTTATGTAAA-3′ for PPM1B, forward 5′-GCTGTGGTGGTGACTGGATTT-3′ and reverse 5′-GCCCAACTTTCCCAATTCCT-3′ for PGPEP1, forward 5′-GGTATTAGTACTGCCGCCCTC-3′ and reverse 5′-CATTCATTGTGGGCTCCGTG-3′ for TFG, forward 5′-CTATCCCCGCCAAACATCT-3′ and reverse 5′-CCATCTCAGCAGCTTCCTTC-3′ for EF1α^56^. All the primer pairs amplify fragments from cDNA of adjacent exons of the target genes, respectively.

### ChIP assay

ChIP assay on tadpole intestine was done as described previously^57^ with anti-TR (new PB), which recognized both TRα and TRβ in both *Xenopus laevis* and *Xenopus tropicalis* ^24, 58^. All ChIP experiments were done at least twice with similar results.

For immunoprecipitation, the DNA concentration of the chromatin was diluted to 10 ng/μl with ChIP dilution buffer (Millipore). After precleaning with salmon sperm DNA/protein A-agarose beads, input samples were taken, and 500 μl of each chromatin sample were immunoprecipitated with indicated antibodies and salmon sperm DNA/protein A-agarose beads. The mixtures were incubated overnight at 4 °C followed by spinning down the beads. The beads were washed with ChIP buffer I, ChIP buffer II, ChIP buffer III and TE (Millipore). After the last wash, 200 μl of elution buffer were added to the samples as well as the input controls and incubated at 65 °C overnight, and the immunoprecipitated DNA was purified. The DNA was then analyzed by qPCR on an ABI 7000 (Applied Biosciences) with the gene-specific primers. For the detection of exon 5 of *Xenopus tropicalis* TRβ, forward primer 5′-CCCCGAAAGTGAAACTCTAACTCT-3′, reverse primer 5′-CCACACCGAGTCCTCCATTTT-3′ and FAM-labeled Taqman probe CTGCCATCTCACCATTC were used. The following primers were used for the detection of the newly identified TRE regions with SYBR Green detection, forward primer 5′-ATCCCGCCTACTCTTTATTCCTCCAGCTGC-3′, reverse primer 5′-GAGAGAGAGTCAGTGTGGTGGTGGGTCAGA-3′ for MBD3; forward primer 5′-AGTTGTTTGTGTGACCTCGGCCTCA-3′, reverse primer 5′-GCCCTCTGCTCATTTAACTCACAACGGT-3′ for PPM1B; and forward primer 5′-ACGTTCTCCGCTCGCTTCCTTCGGA-3′, reverse primer 5′-TCCGGTGCATGTCAGGACAAGGGCA-3′ for PGPEP1.

The ChIP signals were expressed as the percentage of the total input DNA prior to immunoprecipitations.

### ChIP-on-chip assay

The genomic sequence information for *Xenopus tropicalis* (genome release version 4.1) was used to generate a tiled genomic probe set (479459 probes) covering the sequences from 5.5 kb upstream to 2.5 kb downstream of the predicted transcriptional start site of 17,355 *X. tropicalis* genes (based on Ensembl release 46). This resulted in a probe-set that has about 40 probes (60-mer) tiled for each of the putative promoter regions at an average tiling distance of 205 bp. The Agilent microarray 244 k design was used for this and the genomic array probe-set contains 2 microarray slides (244 k each) for the complete coverage including control probes.

*Xenopus tropicalis* tadpole groups of 20 at stage 54 were treated with either 10 nM T3 for 48 h (treated group) or 0.1X MMR buffer (control group). Each of the groups was duplicated. They were not fed during this treatment. Chromatin immune-precipitation assays with antibody specific to TR (anti-TR(PB)) were performed above^57^. The enriched ChIP DNA was amplified by ligation mediated PCR (LM-PCR) using Agilent supplied adaptors, labeled with Cy5. The total genomic DNA isolated prior to antibody immunoprecipitation was similarly labeled with Cy3 (Input). Both Cy3 and Cy5 labeled DNAs were, hybridized to the microarray, washed, scanned, features extracted and primary scan data analyzed according to manufacturer’s instructions (Agilent Mammalian ChIP-on-chip, version 9.2).

### Transcription assay in *Xenopus laevis* oocytes

The plasmids containing GFP (green fluorescent protein), *Xenopus laevis* TRα and RXRα were linearized and transcribed *in vitro* using an mMESSAGE mMACHINE SP6 Transcription kit (Ambion). The cytoplasm of stage VI *Xenopus laevis* oocytes was injected with totally 46 pg per oocyte of the green fluorescent protein (GFP) mRNA or TR and RXR mRNAs. Two hours later, the firefly luciferase reporter construct (345 pg per oocyte) and the control phRG-TK *Renilla* luciferase (Promega) (34.5 pg per oocyte) were co-injected into the oocyte nucleus. After overnight incubation at 18 °C in the presence or absence of 100 nM T3, the injected oocytes were prepared for luciferase assay by using the Dual-Luciferase Reporter Assay system according to the manufacture’s protocol (Promega). Three oocytes per sample were lysed in 45 μl of 1X Passive buffer (Promega), and 10 μl of lysate were used for the luciferase assays. Three independent samples were used for each injection at the same time. The relative expression level of firefly luciferase to *Renilla* luciferase was determined. Each data point represents the average of the 5 samples with the standard error.

### Bioinformatics analyses

The ChIP-on-Chip data were analyzed to identify peaks of TR binding in 8 kb promoter region for each gene (Personal Diagnostix Inc., Gaithursburg, MD). The data were pretreated by removing low quality probes that were flagged in the ChIP-on-chip analysis with statistical analysis using R package Limma. In brief, background intensities were subtracted from the ChIP intensities and within array normalization was carried out with Loess normalization to balance M (log-ratios) and A (average intensities)-values. Normalization was performed between arrays of the same treatment to ensure that A-values had the same empirical distribution across arrays leaving the M-values unchanged. Average intensities for the same treatment were calculated and smoothened by using a sliding window of genomic region of 1400 bp. The enriched regions of binding events were identified with the criterion of at least 3 consecutive probes having ChIP signal intensities of more than 2.5 times of standard deviation. The identities of putative promoters on the ChIP-on-chip microarray slides were determined based on their ENSEMBL identification numbers.

## Results

### Identification of TR-binding sites in premetamorphic intestine by ChIP-on-chip assay

**Figure 1:**
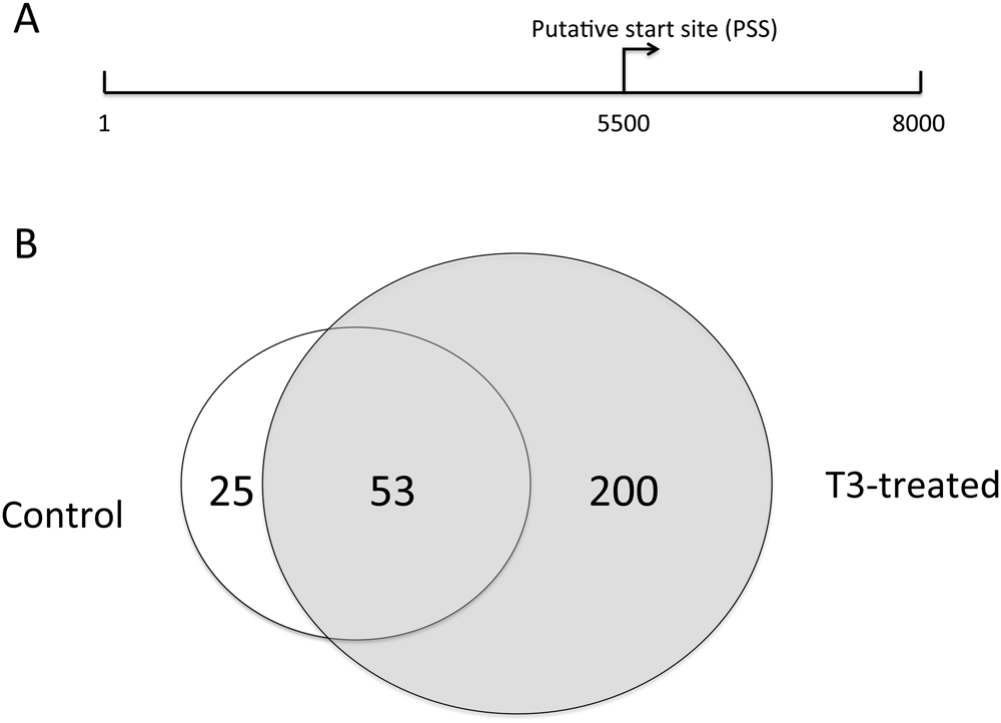
ChIP-on-chip assay identified putative TR targets in the tadpole intestine. (**A**) Schematic representation of a gene in the genomic chips. About 5500 bp upstream and 2500 bp downstream of the 5′-end of the cDNA sequence in the databank for each putative gene in the *Xenopus tropicalis* genome database were used to design a 60 bp oligonucleotide probe at an average tiling distance of 205 bp, covering the entire 8000 bp. The probes were custom-printed onto the genomic chips. PSS: putative transcription start site. (**B**) Venn-diagram showing the genes with TR binding sites as identified from the ChIP-on-chip assay with the intestine samples from control and T3-treated stage 54 premetamorphic tadpoles. Note that vast majority of the genes bound by TR in the control tadpoles were also found in the T3-treated animals.

**Figure 2:**
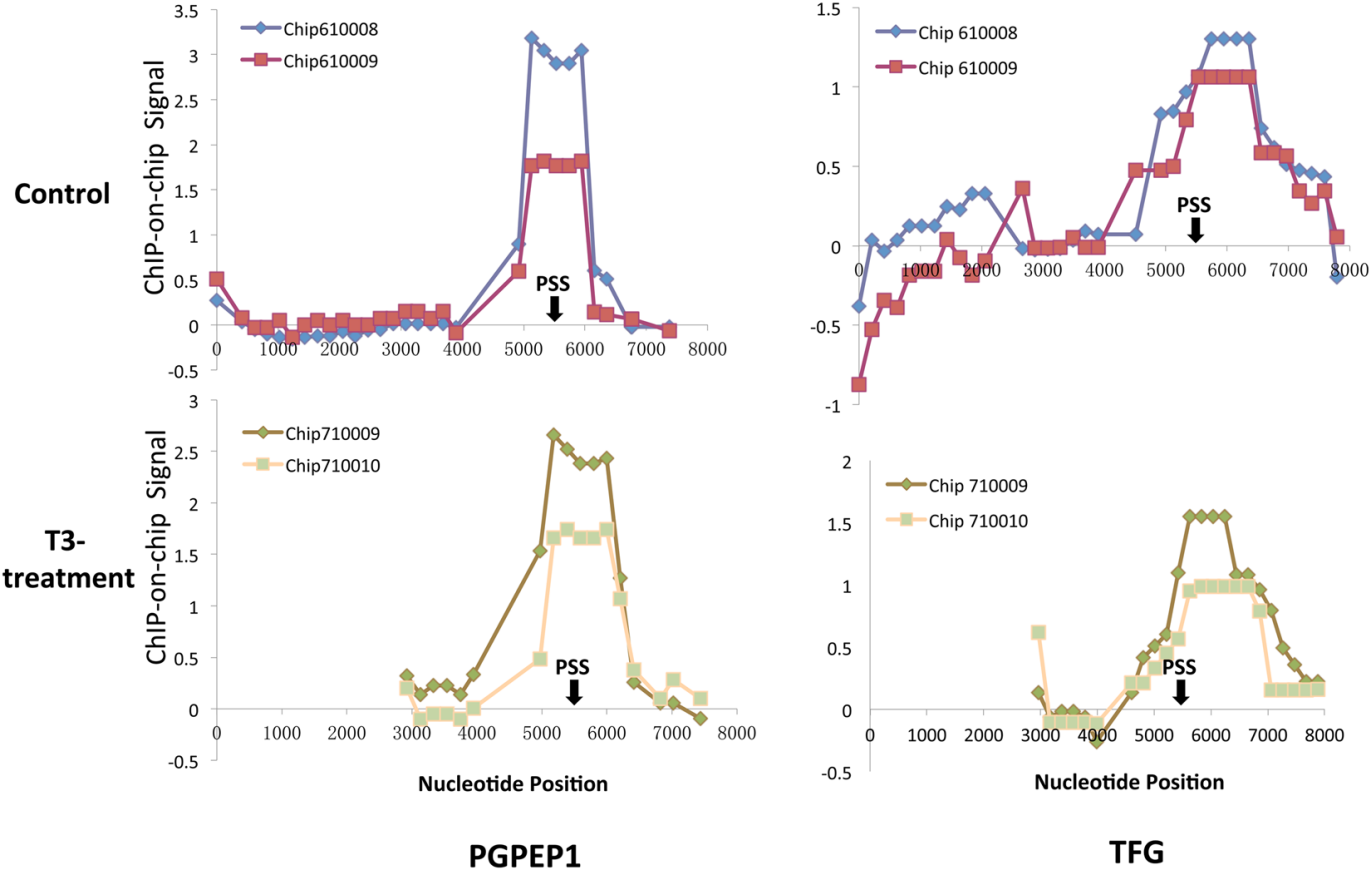
The ChIP signals across the 8000 bp promoter region for PGPEP1 and TFG1 from the control and T3 treated tadpole intestine. Both the control and T3-treated groups had two independent samples as shown. The arrow points to the putative start site (PSS).

A genomic microarray chip set consisting of two chips was generated to cover the sequences from 5.5 kb upstream to 2.5 kb downstream of the 5′-end of the about 17,355 known and predicted *Xenopus tropicalis* genes in the genomic database (Fig. 1A). Each gene had 40 probes of 60 bases in length (60-mer) at about 205 bp apart, spanning the entire 8 kb of the genomic sequence. To identify genes that are bound by TR in the intestine, we treated premetamorphic, stage 54, *Xenopus tropicalis* tadpoles with 10 nM T3 for 2 days. The intestine was isolated from the control and T3 treated tadpoles and subjected to anti-TR antibody ChIP-on-chip assay by using genomic microarray chip set. Two duplicated samples were done for each of the control and T3-treated groups. Initial analysis of the ChIP-on-chip data by aligning the ChIP signals to the partially annotated *Xenopus tropicalis* genomic scaffolds identified very limited number of candidate TR-bound genes, likely due to the poor annotation (data not shown). Thus, we next analyzed the ChIP-on-chip data against only the tiling sequences from 5.5 kb upstream to 2.5 kb downstream of the 5′-end of the 17,355 genes used for preparing the genomic microarray chips. The analyses led to the identification of 78 genes with significant TR binding in the control tadpole intestine and 253 genes in the T3-treated tadpole intestine, with 53 genes identified in both the control and T3-treated group (Figs 1 and 2) (Supplemental Table 1).

**Figure 3:**
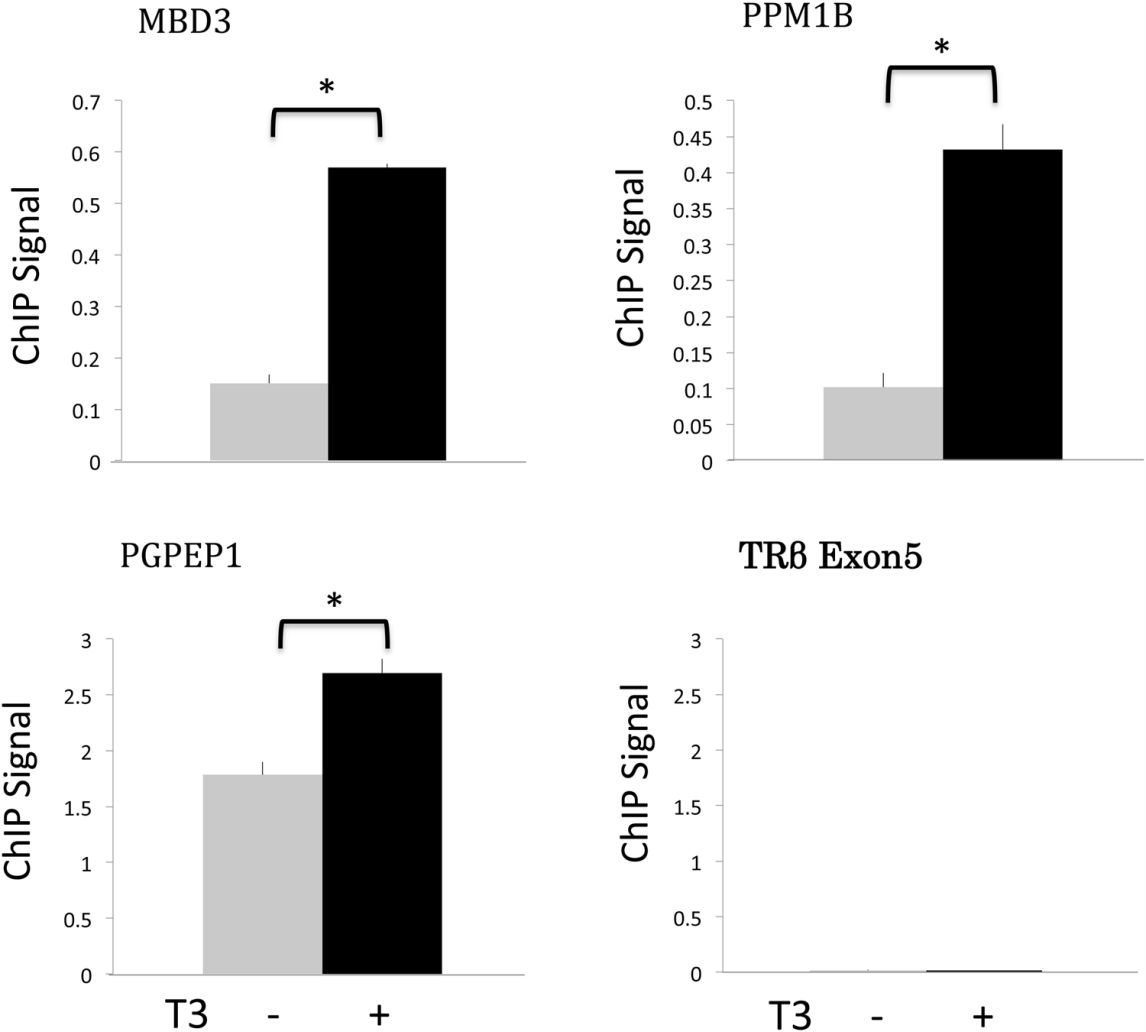
ChIP confirmation of the TR binding to target sites identified from the ChIP-on-chip assay. The intestine from stage 54 tadpoles treated with or without T3 was isolated and subjected to anti-TR antibody ChIP assay and the region around the putative TR binding sites as identified from the ChIP-on-chip assay was PCR amplified. Note that no TR binding was found in the control gene (exon 5 of the TRβ gene) while all three newly identified target genes had TR binding in the absence of T3 and this binding was enhanced upon T3 treatment, in agreement with the ChIP-on-chip data. * indicates pairs of samples with significant differences (p < 0.05).

To validate the ChIP-on-chip findings independently, we selected 8 genes (Dot1L, MBD3, PPM1B, PGPEP1, JUNB, BEND7, PUM2, and TGFα, see Supplemental Table 1) based on the analyses of ChIP signals against both the genomic scaffolds and the sequences used for preparing the genomic microarray chips as well as the presence of putative TREs within the tiling sequences from a bioinformatics analysis (see below). We designed two PCR primers around the putative TRE(s) for each gene and carried out regular ChIP assays by using the TR antibody and the intestine from stage 54 premetamorphic *Xenopus tropicalis* tadpoles treated with or without 10 nM T3 for 2 days. The results showed that all 8 genes analyzed, including the MBD3 (Methyl-CpG binding domain protein 3), PPM1B (Protein phosphatase, Mg2+/Mn2+ dependent 1B) and PGPEP1 (Pyroglutamyl-Peptidase I), were indeed bound by TR in the intestine (Fig. 3 and data not shown).

### Regulation of TR target genes by T3 in the intestine

**Figure 4:**
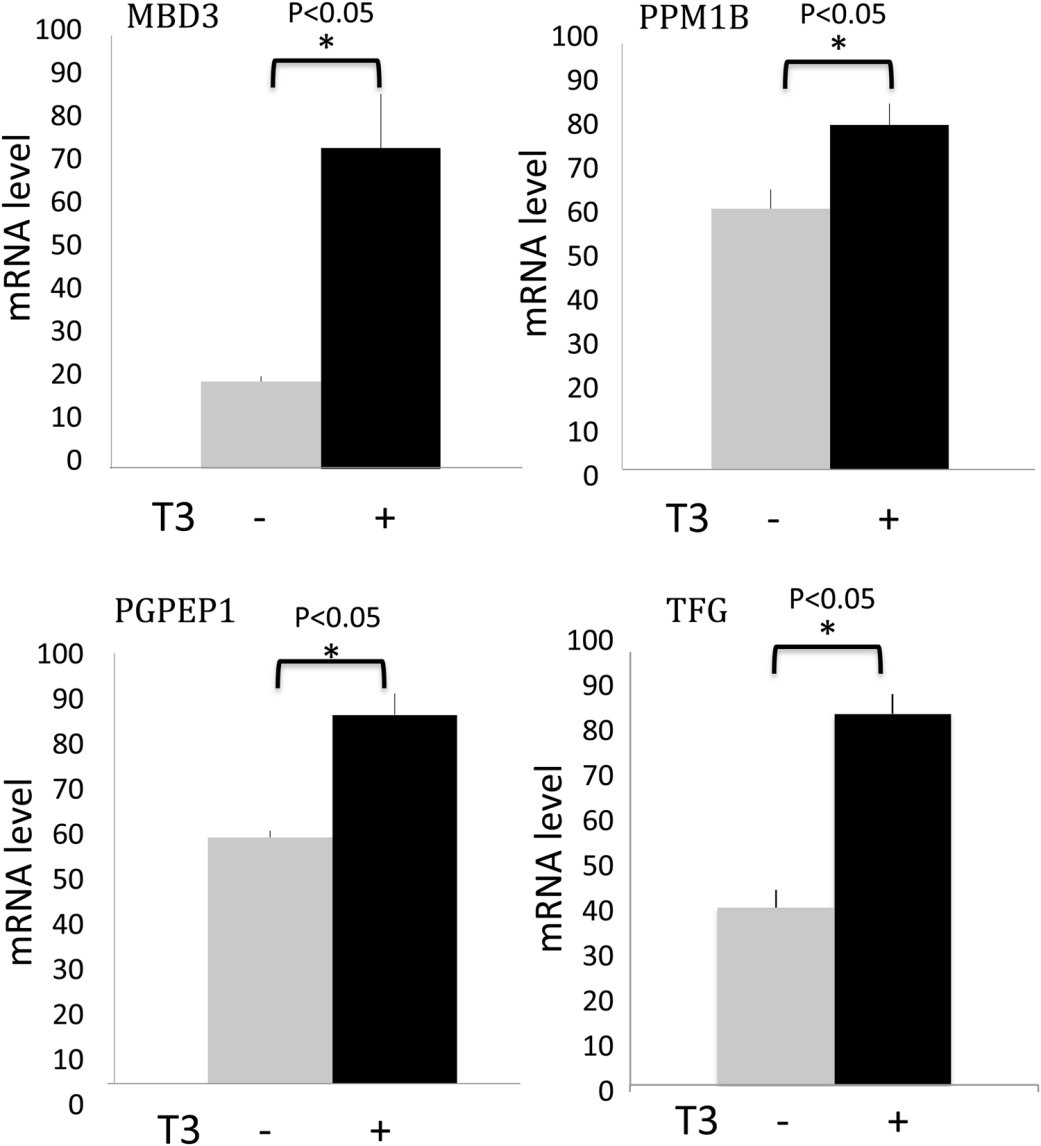
RT-PCR analysis confirming the regulation of newly identified TR targets as T3 response genes. The RNA was isolated from the intestine of stage 54 tadpoles treated with or without T3 was isolated and subjected to RT-PCR analysis for gene expression. Note that all three genes were found to be induced by T3 treatment. *Indicates pairs of samples with significant differences (p < 0.05).

To determine if the newly identified candidate TR target genes are regulated by T3 in the tadpole intestine, stage 54 premetamorphic *Xenopus tropicalis* tadpoles were treated with or with 10 nM T3 for 2 days and total RNA was isolated from the intestine. The expression of the 8 genes validated by ChIP assay above was determined by qRT-PCR. Of the 8 genes analyzed, 6 genes were found to be induced by T3 in the tadpole intestine (Fig. 4 and data not shown), suggesting that most of the newly identified genes bound by TR were T3 response genes. The expression levels of the other two were too low to determine their regulation by T3. It is possible that these two genes are still T3-regulated genes *in vivo* or under different treatment conditions.

### The TR target genes contain functional TREs

**Figure 5:**
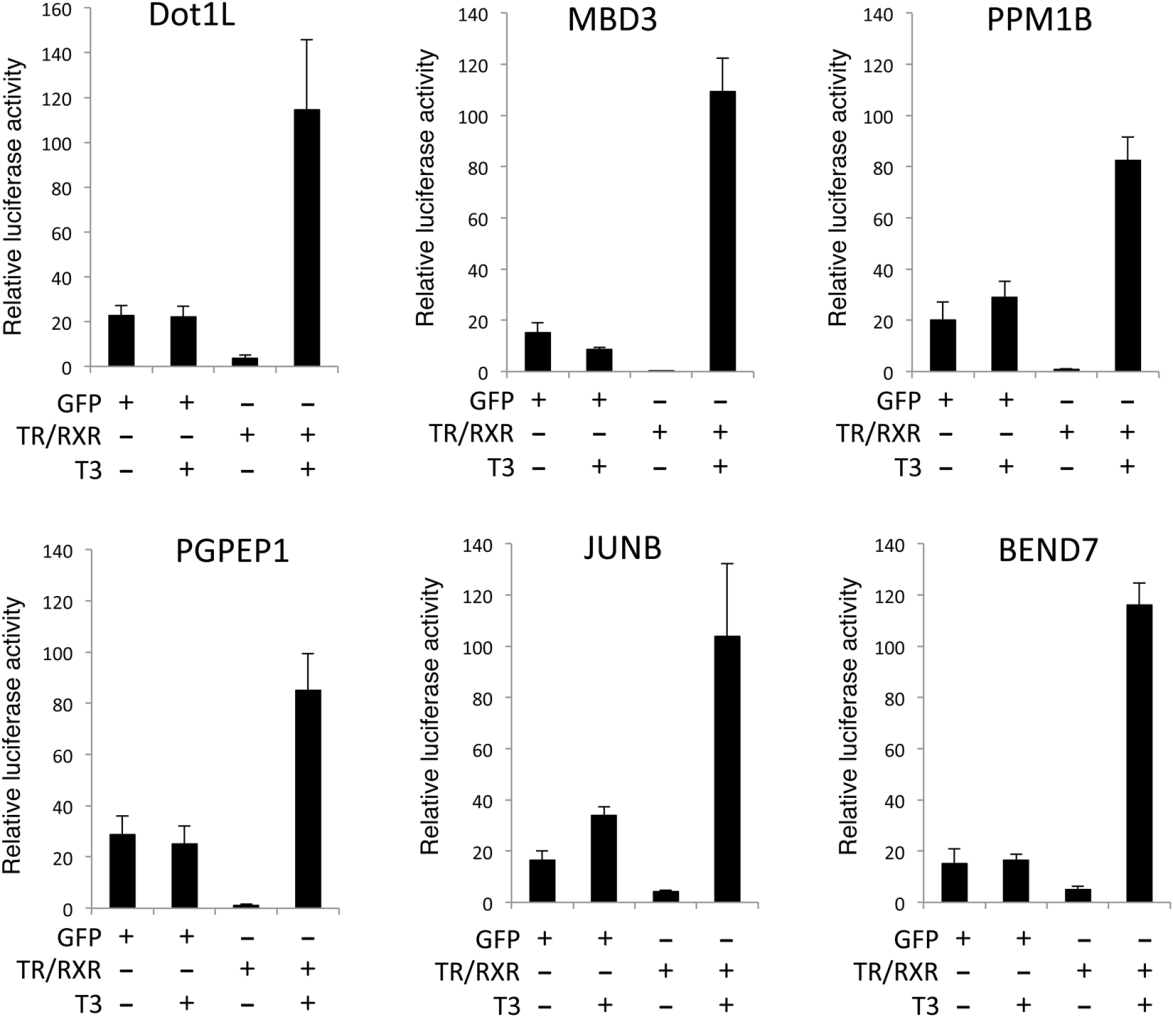
The putative TREs in the candidate TR target genes can mediate transcriptional activation by T3 in frog oocytes. The luciferase reporter construct containing the TREs of Dot1L, MBD3, PPM1B, PGPEP1, JUNB, BEND7, respectively, was co-injected with the control *Renilla* luciferase construct phRG-TK into the nuclei of *Xenopus* oocytes with or without prior cytoplasmic injection of *Xenopus laevis* TRα and RXRα mRNAs or GFP mRNA as negative control. The oocytes were incubated at 18 °C overnight in the presence or absence of 100 nM T3 and then used for dual luciferase assays. The relative activities of the firefly luciferase to *Renilla* luciferase were plotted. Note that all the reporters responded to T3 in the presence of TR/RXR.

Among the 278 TR target genes identified by ChIP-on-chip assay, 191 genes had 1 or more putative TRE, as revealed by NHR SCAN analysis (http://www.cisreg.ca/cgi-bin/NHR-scan/nhr_scan.cgi), within the 8 kb sequences covered on the ChIP-on-chip slides (Data not shown). To investigate if these TREs were functional TREs, we chose several TREs from the 8 selected genes whose binding by TR was confirmed (Fig. 3 and data not shown) and analyzed their function in the reconstituted *Xenopus laevis* oocyte transcription system, where the reporter DNA is chromatinized^24, 58^. It is well-known that in this system or in tissue culture transfection studies, TREs that are located about 1 kb or further away from the site has little effect on the promoter activity^59^. Thus, we test the function of the putative TREs in a heterologous reporter. The *Xenopus tropialis* Dot1L promoter has been shown to be regulated by TR during *Xenopus* development^53^, and it is also among the candidate target genes identified by our ChIP-on-chip assay. The Dot1L promoter was cloned to drive firefly luciferase gene expression in frog oocytes and showed to be regulated directly by T3 (Fig. 5). This T3-regulation of the Dot1L promoter is mediated by a functional TRE in the proximity of transcriptional start site and mutation within the TRE sequences abolished T3-regulation on the promoter^53^. Importantly, placing the putative TREs from the selected genes in place of the Dot1L TRE also enabled the promoter to be activated by T3 in the presence of TR/RXR (Fig. 5), suggesting that they are likely functional TREs mediating the response of the endogenous genes to T3.

### Many of the newly identified TR-bound genes are regulated during intestinal metamorphosis

**Figure 6:**
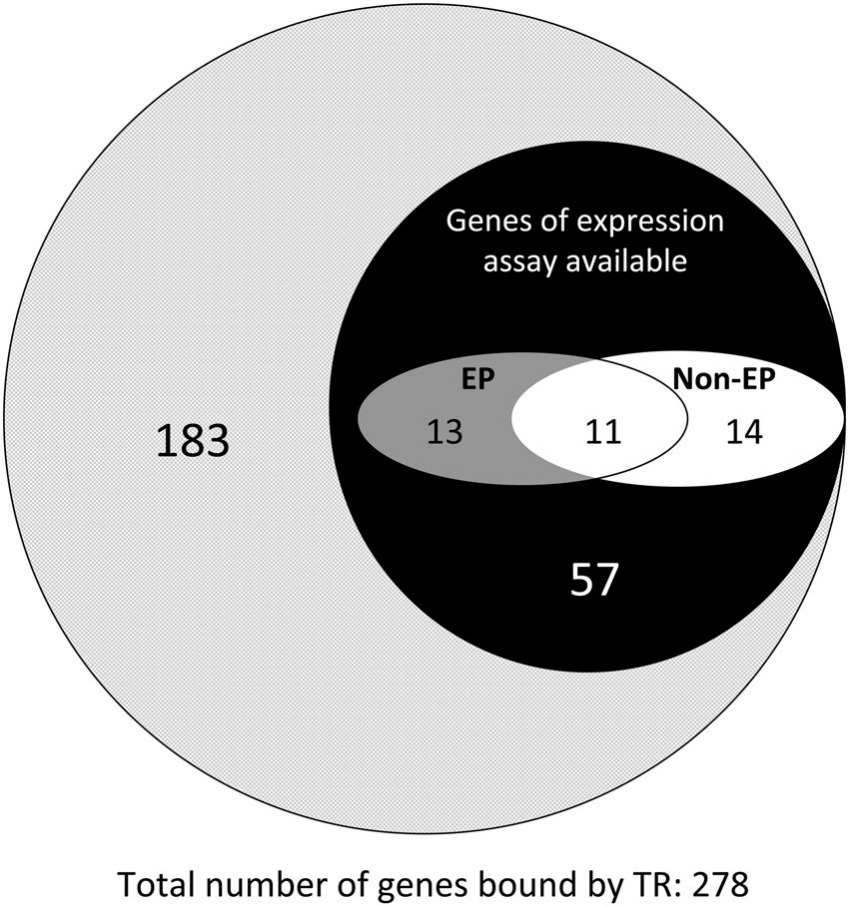
Venn diagram showing overlap of the TR target genes identified by ChIP-on-chip assay with known developmentally regulated genes identified from an early expression microarray study. Of the 278 TR target genes identified by ChIP-on-chip assay, 95 or 34% were also found on the microarray used for the expression study (Note that the actually overlap might be higher as most genes may have different names on the microarray chips used in the expression study and on the genomic chips used here, therefore showing up as non-overlapping). Of the 95 genes present in the gene expression microarray, 38 were found to be regulated during metamorphosis in the intestine in either the epithelium (EP, 13 genes), non-epithelium (Non-EP, 14 genes) or both (11 genes), suggesting that about 40% of the genes identified here are regulated by T3 during intestinal metamorphosis.

We have previously carried out a gene expression microarrays to identify genes whose expression is altered in the intestinal epithelium (EP) or the non-epithelium (Non-EP, or the rest of the intestine) during natural metamorphosis in *Xenopus laevis* ^43^, a highly related species whose metamorphosis resembles that in *Xenopus tropicalis* with essentially identical morphological and molecular properties^24, 53, 60–63^. Of the 278 TR target genes identified by ChIP-on-chip assay, 95 or 34% are also found on the *Xenopus laevis* microarray chips used for the expression study (Note that the actually overlap might be much higher as most genes may have different names on the microarray chips used in the expression study vs. the genomic chips here, therefore showing up as non-overlapping). Of these 95 genes present in the gene expression microarray, 38 genes or 40% were found to be regulated during metamorphosis in the intestine in either the epithelium (EP), non-epithelium (Non-EP) or both (Fig. 6) (Supplemental Table 1). As the microarray studies were done with only 3 developmental stages, it is very likely that many developmentally regulated genes were not identified by the microarray analysis. Thus, it is possible that most of the genes identified here are regulated in the intestine during metamorphosis, as suggested by the above RT-PCR expression analysis of selected genes in the intestine of premetamorphic tadpoles treated with or without T3.

## Discussions and Conclusions

T3 is known to regulates gene transcription by binding to TRs, which bind to T3 response elements (TREs) in their target genes as either homodimers or heterodimers formed with 9-cis retinoic acid receptors (RXRs)^1, 2, 64, 65^. Many T3 response genes have been identified over the years by using various approaches in cell cultures and *in vivo*, relatively few have been shown to be direct TR target genes *in vivo*, especially during vertebrate development. Here, using an unbiased genome-wide approach, we have identified nearly 300 genes bound by TR in the premetamorphic tadpole intestine and provided evidence to support the involvement of these binding sites in mediating the regulation of these genes by T3 during intestinal metamorphosis.

The intestinal metamorphosis has been used as a model to investigate the *in vivo* mechanism of gene regulation by TR during vertebrate development. The vertebrate intestine is one of the best-studied organs where self-renewal is an integral part of their physiological function^66, 67^. The establishment and/or maturation of this self-renewal system in all vertebrates takes place during the postembryonic period when plasma T3 concentrations are high^4, 68–71^. In amphibians such as *Xenopus laevis* and *Xenopus tropicalis*, this takes place during metamorphosis and involves T3-dependent apoptotic degeneration of most of the larval epithelial cells and concurrent *de novo* formation of adult epithelial stem cells via dedifferentiation of some larval cells through yet-unknown mechanism^67, 72–81^. This makes intestinal metamorphosis an excellent model system to study the formation of adult organ-specific stem cells during vertebrate development.

Earlier studies have shown that TR plays an essential role in regulating premetamorphic development and mediating T3-effect on metamorphosis^8, 22, 23, 25–34, 36, 37^. T3 is believed to induce a gene regulation cascade that is responsible for metamorphic transformation of individual organs/tissues and the immediate early T3 response genes, i.e., those regulated by T3 directly at the transcription level when T3 first becomes available, are expected to play critical roles in this cascade. Toward understanding how T3 induces the formation of adult intestinal stem cells, many T3 response genes have been isolated over the years by using various means^39, 43–45^. However, few of the genes identified so far are shown to be direct TR target genes *in vivo*. On the other hand, our studies suggest that most genes that we have identified here are such immediate early T3 response genes regulated by TR at the transcription level. First, by definition, all genes identified from our ChIP-on-chip assay are bound by TR in the intestine either directly or indirectly, a prerequisite of immediate early T3 response genes. Of the 8 genes that we chose to validate the TR binding by traditional ChIP assay, all were confirmed to be bound by TR in the intestine. Second, most of these 8 genes (6 out 8) were indeed found to be regulated in the intestine when premetamorphic tadpoles were treated with T3 with the expression of the remaining two too low to be determined. Third, by comparing to the previously published developmental tissue-specific microarray, we found that 40% of the genes identified here were regulated in either the epithelium, non-epithelium, or both in the intestine during T3-dependent metamorphosis, indicating that these genes are indeed regulated by T3 during natural metamorphosis. Considering the limited stages (only 3 of the 10 between stages 56–66) used in the intestinal expression microarray study^43^, it is possible that many other genes are also regulated in the intestine at some stages of metamorphosis. Finally while the ChIP-on-chip data is being analyzed, we characterized the promoter of one of the newly discovered TR-bound genes, the Dot1L gene, and indeed found it to contain a functional TRE near the transcription start site and mutating the functional TRE in the Dot1L promoter abolished T3-activation on the promoter^53^. Furthermore, replacing the functional TRE with the putative TREs in several other genes discovered from our ChIP-on-chip assay also enabled the promoter to respond to T3 *in vivo* (Fig. 5). It is worth pointing out that such an assay does not exactly test if the TRE is functional in the context of its own *in situ* location in the genome. Such a test would require future studies such as mutagenizing the endogenous TRE in the genome and studying the effect *in vivo*. On the other hand, a promoter fragment containing a TRE similarly identified and characterized for another candidate gene discovered here, histidine ammonia-lyase 2, could drive T3-inducible, transgenic GFP expression in the intestinal stem cells during metamorphosis, mimicking the endogenous promoter^55, 78^. Regardless, all these together suggest that the genomic regions bound by TR as identified from our ChIP-on-chip assay are likely involved in regulating the corresponding genes during development.

Recent advancements in genome-wide chromatin immunoprecipitation analyses have led to identification of genes bound by TR *in vivo* in mouse cell cultures or organs. A few studies used ChIP-seq for unbiased analyses of genome-wide TR binding sites in mouse cell cultures^82^ and adult liver^83, 84^. These studies led to the identification of thousands of TR binding sites. Many of the identified binding sites are located far away for the nearest genes and thus their roles in T3-dependent gene regulations are unknown and difficult to investigate. Another study employed ChIP-on-chip to analyze TR binding sites in the developing mouse cerebellum^85^. Here, the authors used a genomic chip containing −8 kb to +2 kb around the transcription start site of 5000 mouse genes and identified 91 genes with TR bindings sites. They confirmed 10 out of 13 binding regions. However, the regulation of these genes by T3 was largely unclear as the authors only analyzed the expression of 4 of the 10 validated genes. Our studies here thus represent the first comprehensive genome-wide analysis of TR target genes during development *in vivo* where most of the newly identified TR target sites were confirmed and/or found to be regulated by T3 and/or T3-dependent intestinal remodeling. Although the use of genomic chips for microarray left out many genomic regions, especially the large intergenic regions, leaving many other potential TR binding sites undiscovered, the close-proximity of the sites that we have discovered to the transcription start site suggest that the TR bindings sites identified here are indeed responsible for the regulation of the genes by T3 during development. This is indeed supported by the analysis of the promoter of one of the genes, the Dot1L gene, as indicated above. In addition, even with the recent progresses in genome-annotation in *Xenopus troplicalis* ^86^, the poor status of the genome assembly and, annotation in *Xenopus troplicalis* makes it very difficult to carry out genome-wide ChIP-seq analysis as recently discussed by Grimaldi *et al*.^60^. In any case, the identification of the direct target genes here should facilitate future studies on the mechanisms of tissue-specific gene regulation by TR since many genes, like the HAL2^55, 78^, are regulated in a tissue-specific manner even within a single organ, and more importantly, on how T3 regulates the formation of adult intestinal stem cells during metamorphosis.

## Electronic supplementary material

Supplemental Table 1
