# Supplementary material for: Genome-wide identification of thyroid hormone receptor targets in the remodeling intestine during Xenopus tropicalis metamorphosis
Source: Sci Rep. 2017 Jul 25;7:6414. doi: 10.1038/s41598-017-06679-x (PMC5527017; doi:10.1038/s41598-017-06679-x)
Supplement: Supplementary file 1 — Supplemental Table 1 [file 41598_2017_6679_MOESM1_ESM.pdf]

Genome-wide identification of thyroid hormone receptor targets in the remodeling intestine during *Xenopus tropicalis* metamorphosis

Liezhen Fu, Biswajit Das, Kazuo Matsuura, Kenta Fujimoto, Rachel A. Heimeier, and Yun-Bo Shi

Section on Molecular Morphogenesis, Eunice Kennedy Shriver National Institute of Child Health and Human Development (NICHD), National Institutes of Health (NIH), Bethesda, Maryland, 20892

***Corresponding Authors**: Yun-Bo Shi, Shi@helix.nih.gov

Supplemental Table 1. Genes bound by TR in the intestine and its regulation during development

ENSEMBLE ID	Peak signal	Peak position	Gene ID	Bound by TR in	Regulated during development in*	Gene Description
ENSXETG00000000089	0.815343925	5946	acy1.1	T3-treatment		aminoacylase 1, gene 1 [Source:Jamboree;Acc:XB-GENE-972681]
ENSXETG00000000161	0.66053772	5946	sae1	T3-treatment		SUMO1 activating enzyme subunit 1 [Source:Jamboree;Acc:XB-GENE-923356]
ENSXETG00000000254	0.572419037	5536	gtdc1	Control		glycosyltransferase-like domain containing 1 [Source:Jamboree;Acc:XB-GENE-1004420]
ENSXETG00000000559	0.968453088	5536	rcbtb2	T3-treatment		regulator of chromosome condensation (RCC1) and BTB (POZ) domain containing protein 2 [Source:Jamboree;Acc:XB-GENE-952598]
ENSXETG00000000572	1.511153686	5126	acvr1b	T3-treatment	Both EP & Non-EP	activin A receptor, type IB [Source:Jamboree;Acc:XB-GENE-483904]
ENSXETG00000000579	0.716015803	411	nr4a1	T3-treatment		nuclear receptor subfamily 4, group A, member 1 [Source:Jamboree;Acc:XB-GENE-483315]
ENSXETG00000000804	0.575752145	4511	nfil3	Control	Both EP & Non-EP	nuclear factor, interleukin 3 regulated [Source:Jamboree;Acc:XB-GENE-479624]
ENSXETG00000000835	0.589051682	821	nfib	T3-treatment		nuclear factor I/B [Source:Jamboree;Acc:XB-GENE-486376]
ENSXETG00000001582	0.679877262	2666	spata5	Control		spermatogenesis associated 5 [Source:Jamboree;Acc:XB-GENE-6054426]
ENSXETG00000001921	0.728222458	2666	setd1a	T3-treatment		SET domain containing 1A [Source:Jamboree;Acc:XB-GENE-6046043]
ENSXETG00000001957	0.64693736	5671	cul4a	T3-treatment		cullin 4A [Source:Jamboree;Acc:XB-GENE-1014775]
ENSXETG00000002040	0.713648802	2461	myo19	T3-treatment		myosin XIX [Source:Jamboree;Acc:XB-GENE-999897]
ENSXETG00000002271	0.576996686	5741	ranbp9	T3-treatment		RAN binding protein 9 [Source:Jamboree;Acc:XB-GENE-494852]
ENSXETG00000002589	0.727347698	6151	rhou	T3-treatment		ras homolog gene family, member U [Source:Jamboree;Acc:XB-GENE-491190]
ENSXETG00000002643	0.67719223	6356	zmym2	T3-treatment		zinc finger, MYM-type 2 [Source:Jamboree;Acc:XB-GENE-961591]
ENSXETG00000002746	0.583056381	5331	ppm1b	Both		protein phosphatase, Mg2+/Mn2+ dependent, 1B [Source:Jamboree;Acc:XB-GENE-999170]
ENSXETG00000002843	0.72473583	5741	XB-GENE-992854	T3-treatment		Putative ortholog of galectin-4 (Lactose-binding lectin 4) (L-36 lactose binding protein) (L36LBP), 1 of 1 [Source:Jamboree;Acc:XB-GENE-992854]
ENSXETG00000002899	0.633070638	1026	zfp36	T3-treatment		zinc finger protein 36, C3H type, homolog [Source:Jamboree;Acc:XB-GENE-991815]
ENSXETG00000003127	0.642328265	616	ikzf2	T3-treatment		IKAROS family zinc finger 2 (Helios) [Source:Jamboree;Acc:XB-GENE-1013632]
ENSXETG00000003144	0.824862277	1846	gata6	T3-treatment		GATA binding protein 6 [Source:Jamboree;Acc:XB-GENE-485063]
ENSXETG00000003160	0.751999685	411	dbp	T3-treatment		D site of albumin promoter (albumin D-box) binding protein [Source:Jamboree;Acc:XB-GENE-5995286]
ENSXETG00000003203	0.82465633	538	hadhb	T3-treatment		hydroxyacyl-CoA dehydrogenase/3-ketoacyl-CoA thiolase/enoyl-CoA hydratase (trifunctional protein), beta subunit [Source:Jamboree;Acc:XB-GENE-1009673]
ENSXETG00000003291	0.593119427	5331	polr2e	T3-treatment	Non-EP	polymerase (RNA) II (DNA directed) polypeptide E, 25kDa [Source:Jamboree;Acc:XB-GENE-1005304]
ENSXETG00000003440	0.600161789	1846	akap9	T3-treatment		A kinase (PRKA) anchor protein (yotiao) 9 [Source:Jamboree;Acc:XB-GENE-5953770]
ENSXETG00000003506	1.152658914	5536	urod	T3-treatment	Both EP & Non-EP	uroporphyrinogen decarboxylase [Source:Jamboree;Acc:XB-GENE-948493]
ENSXETG00000003539	0.770937274	2461	dydc1	T3-treatment		DPY30 domain containing 1 [Source:Jamboree;Acc:XB-GENE-6258872]
ENSXETG00000003760	0.959061959	821	setd1b	T3-treatment		SET domain containing 1B [Source:Jamboree;Acc:XB-GENE-5842344]
ENSXETG00000004036	0.776047649	4013	ctu1	T3-treatment		cytosolic thiouridylase subunit 1 homolog [Source:Jamboree;Acc:XB-GENE-5872210]
ENSXETG00000004154	0.78070366	5741	cyp20a1	T3-treatment		cytochrome P450, family 20, subfamily A, polypeptide 1 [Source:Jamboree;Acc:XB-GENE-988532]
ENSXETG00000004226	0.890489097	2666		T3-treatment		
ENSXETG00000004328	0.927936434	5126	commd5	Both		COMM domain containing 5 [Source:Jamboree;Acc:XB-GENE-952087]
ENSXETG00000004334	0.63553975	3896	slc25a43	T3-treatment		solute carrier family 25, member 43 [Source:Jamboree;Acc:XB-GENE-6258507]
ENSXETG00000004766	0.669806752	1846	junb	T3-treatment		jun B proto-oncogene [Source:Jamboree;Acc:XB-GENE-945864]
ENSXETG00000005251	0.727856369	5455	tor2a	Both		torsin family 2, member A [Source:Jamboree;Acc:XB-GENE-5806617]
ENSXETG00000005358	0.637732736	2384		T3-treatment		Uncharacterized protein [Source:UniProtKB/TrEMBL;Acc:F6QEX1]
ENSXETG00000005387	0.812597399	5536	pm20d2	Both		peptidase M20 domain containing 2 [Source:Jamboree;Acc:XB-GENE-969482]
ENSXETG00000005620	1.672538267	1231	LONRF1	Both		LON peptidase N-terminal domain and ring finger 1 [Source:HGNC Symbol;Acc:HGNC:26302]
ENSXETG00000005659	0.703968978	5331	impad1	T3-treatment		inositol monophosphatase domain containing 1 [Source:Jamboree;Acc:XB-GENE-5751033]
ENSXETG00000005781	0.701557582	411	btg3	T3-treatment		BTG family, member 3 [Source:Jamboree;Acc:XB-GENE-995111]
ENSXETG00000005902	0.797768876	2666	prtfdc1	Both	Non-EP	phosphoribosyl transferase domain containing 1 [Source:Jamboree;Acc:XB-GENE-942165]
ENSXETG00000005922	0.692352065	5946	hbp1	T3-treatment	Both EP & Non-EP	HMG-box transcription factor 1 [Source:Jamboree;Acc:XB-GENE-950343]
ENSXETG00000006353	0.704171704	3486	pla2g6	T3-treatment		phospholipase A2, group 6 (cytosolic, calcium-independent) [Source:Jamboree;Acc:XB-GENE-920447]
ENSXETG00000006354	0.812978453	5536	kiaa1370	T3-treatment		KIAA1370 [Source:Jamboree;Acc:XB-GENE-5816685]
ENSXETG00000006455	0.614311642	2051	camk2g	T3-treatment	Non-EP	calcium/calmodulin-dependent protein kinase (CaM kinase) II gamma [Source:Jamboree;Acc:XB-GENE-868520]
ENSXETG00000006575	0.698228027	5536	znf420	Control		zinc finger protein 420 [Source:Jamboree;Acc:XB-GENE-5724906]
ENSXETG00000006612	0.57694666	821	asah2	T3-treatment		N-acylsphingosine amidohydrolase (non-lysosomal ceramidase) 2 [Source:Jamboree;Acc:XB-GENE-1003068]
ENSXETG00000006614	1.508829842	1026	sgms1	T3-treatment		sphingomyelin synthase 1 [Source:Jamboree;Acc:XB-GENE-986773]
ENSXETG00000006633	0.888552066	411	znf706	T3-treatment		zinc finger protein 706 [Source:Jamboree;Acc:XB-GENE-980122]
ENSXETG00000006658	0.609945757	3281	dpy19l4	T3-treatment		dpy-19-like 4 [Source:Jamboree;Acc:XB-GENE-6034528]
ENSXETG00000006692	0.624332706	5946		Both		
ENSXETG00000006970	0.696381336	4716	jup	T3-treatment	Non-EP	junction plakoglobin [Source:Jamboree;Acc:XB-GENE-489397]
ENSXETG00000006990	1.434873376	411		Both		
ENSXETG00000006993	0.760743505	7278		Both		
ENSXETG00000007013	0.867530501	5331	klf11	T3-treatment	EP	Kruppel-like factor 11 [Source:Jamboree;Acc:XB-GENE-956858]
ENSXETG00000007026	1.579606243	5536	prkaa1	T3-treatment		protein kinase, AMP-activated, alpha 1 catalytic subunit [Source:Jamboree;Acc:XB-GENE-946195]
ENSXETG00000007200	0.664909868	1641	haghl	T3-treatment		hydroxyacylglutathione hydrolase-like [Source:Jamboree;Acc:XB-GENE-987560]
ENSXETG00000007220	0.748116237	5126	rps2	T3-treatment		ribosomal protein S2 [Source:Jamboree;Acc:XB-GENE-1003616]
ENSXETG00000007319	1.198831016	5741	c20orf3	T3-treatment		chromosome 20 open reading frame 3 [Source:Jamboree;Acc:XB-GENE-5754317]
ENSXETG00000007320	0.750346854	6356	sema3b	T3-treatment		sema domain, immunoglobulin domain (Ig), short basic domain, secreted, (semaphorin) 3B [Source:Jamboree;Acc:XB-GENE-921561]
ENSXETG00000007329	0.733347816	4921	hyal1	T3-treatment		hyaluronoglucosaminidase 1 [Source:Jamboree;Acc:XB-GENE-987118]
ENSXETG00000007405	0.66533038	5741	dfna5	T3-treatment		deafness, autosomal dominant 5 [Source:Jamboree;Acc:XB-GENE-983245]
ENSXETG00000007460	0.824158955	5331	xpnpep2	T3-treatment		X-prolyl aminopeptidase (aminopeptidase P) 2, membrane-bound [Source:Jamboree;Acc:XB-GENE-969249]
ENSXETG00000007575	0.591146631	411	hoxd13	T3-treatment		homeobox D13 [Source:Jamboree;Acc:XB-GENE-482320]
ENSXETG00000007594	0.734086198	5414	mrpl46	Control	EP	mitochondrial ribosomal protein L46 [Source:Jamboree;Acc:XB-GENE-970912]
ENSXETG00000007619	0.758878187	3691	plekhg1	T3-treatment		pleckstrin homology domain containing, family G (with RhoGef domain) member 1 [Source:Jamboree;Acc:XB-GENE-6050489]
ENSXETG00000007686	0.565087616	5536	fhod1	T3-treatment	EP	formin homology 2 domain containing 1 [Source:Jamboree;Acc:XB-GENE-948719]
ENSXETG00000007744	0.771914651	4809	tmem185a	Both		transmembrane protein 185A [Source:Jamboree;Acc:XB-GENE-972141]
ENSXETG00000007793	0.933356484	6151	abhd11	T3-treatment		abhydrolase domain containing 11 [Source:Jamboree;Acc:XB-GENE-952690]
ENSXETG00000007821	0.756808903	5741	anp32a	T3-treatment		acidic (leucine-rich) nuclear phosphoprotein 32 family, member A [Source:Jamboree;Acc:XB-GENE-982550]
ENSXETG00000007900	0.703378252	4306	slc25a48	T3-treatment		solute carrier family 25, member 48 [Source:Jamboree;Acc:XB-GENE-5770986]

ENSXETG00000008000	0.695937	3281	hnf1b	T3-treatment		HNF1 homeobox B [Source:Jamboree;Acc:XB-GENE-485652]
ENSXETG00000008032	0.742327166	6971	idh1	T3-treatment		isocitrate dehydrogenase 1 (NADP+), soluble [Source:Jamboree;Acc:XB-GENE-1004994]
ENSXETG00000008065	0.623786625	3896	echdc2	T3-treatment		enoyl CoA hydratase domain containing 2 [Source:Jamboree;Acc:XB-GENE-948250]
ENSXETG00000008090	1.344286445	5741	siah1	T3-treatment		seven in absentia homolog 1 [Source:Jamboree;Acc:XB-GENE-1014415]
ENSXETG00000008132	0.905530231	4921	ankrd17	T3-treatment		ankyrin repeat domain 17 [Source:Jamboree;Acc:XB-GENE-994421]
ENSXETG00000008281	0.680314796	3691	isoc1	T3-treatment		isochorismatase domain containing 1 [Source:Jamboree;Acc:XB-GENE-491284]
ENSXETG00000008472	0.58478264	5741	cyct	T3-treatment		cytochrome c, testis [Source:Jamboree;Acc:XB-GENE-5931974]
ENSXETG00000008628	0.60117618	4921	psmc6	T3-treatment		proteasome (prosome, macropain) 26S subunit, ATPase, 6 [Source:Jamboree;Acc:XB-GENE-978099]
ENSXETG00000008843	1.306369732	5741	gpatch8	T3-treatment		G patch domain containing 8 [Source:Jamboree;Acc:XB-GENE-5789711]
ENSXETG00000008880	1.25908231	5331	hook1	Both		hook homolog 1 [Source:Jamboree;Acc:XB-GENE-975863]
ENSXETG00000008901	0.99212961	3486	il4i1	Both		interleukin 4 induced 1 [Source:Jamboree;Acc:XB-GENE-6037585]
ENSXETG00000008903	1.117761716	5741	pum2	T3-treatment		pumilio homolog 2 [Source:Jamboree;Acc:XB-GENE-977105]
ENSXETG00000008931	0.745656389	7381	cers2	T3-treatment		ceramide synthase 2 [Source:Jamboree;Acc:XB-GENE-852703]
ENSXETG00000008966	0.691469657	5126	syap1	Control		synapse associated protein 1 [Source:Jamboree;Acc:XB-GENE-5952691]
ENSXETG00000008968	0.874312543	4829	dtx2	T3-treatment	Non-EP	deltex homolog 2 [Source:Jamboree;Acc:XB-GENE-984000]
ENSXETG00000009117	1.160053062	5946	bend7	T3-treatment		BEN domain containing 7 [Source:Jamboree;Acc:XB-GENE-6039598]
ENSXETG00000009145	0.723849614	411	slc2a2	T3-treatment	Both EP & Non-EP	solute carrier family 2 (facilitated glucose transporter), member 2 [Source:Jamboree;Acc:XB-GENE-486462]
ENSXETG00000009191	0.604202126	4921	trim63	T3-treatment		tripartite motif containing 63 [Source:Jamboree;Acc:XB-GENE-493173]
ENSXETG00000009201	0.582050358	5331	plcxd2	T3-treatment		phosphatidylinositol-specific phospholipase C, X domain containing 2 [Source:Jamboree;Acc:XB-GENE-5832516]
ENSXETG00000009486	0.665686467	6561	smarcc2	T3-treatment		SWI/SNF related, matrix associated, actin dependent regulator of chromatin, subfamily c, member 2 [Source:Jamboree;Acc:XB-GENE-490675]
ENSXETG00000009635	1.33168876	5737	letmd1	Both		LETM1 domain containing 1 [Source:Jamboree;Acc:XB-GENE-962658]
ENSXETG00000009655	0.939018446	5126	mettl1	T3-treatment		methyltransferase like 1 [Source:Jamboree;Acc:XB-GENE-1000662]
ENSXETG00000009865	0.625909864	5331	vdac2	T3-treatment		voltage-dependent anion channel 2 [Source:Jamboree;Acc:XB-GENE-954839]
ENSXETG00000009924	0.716819429	3760		Both		
ENSXETG00000009990	0.619128514	411	mospd2	T3-treatment		motile sperm domain containing 2 [Source:Jamboree;Acc:XB-GENE-957659]
ENSXETG00000010012	0.530055485	5741	sec24d	Control		SEC24 family, member D [Source:Jamboree;Acc:XB-GENE-1003364]
ENSXETG00000010201	1.341229632	6089	bre	T3-treatment		brain and reproductive organ-expressed (TNFRSF1A modulator) [Source:Jamboree;Acc:XB-GENE-1032920]
ENSXETG00000010259	0.570132543	5946	csnk1g3	T3-treatment		casein kinase 1, gamma 3 [Source:Jamboree;Acc:XB-GENE-479074]
ENSXETG00000010510	0.639871586	5741	tnfrsf10b	T3-treatment		tumor necrosis factor receptor superfamily, member 10b [Source:Jamboree;Acc:XB-GENE-941760]
ENSXETG00000010654	0.594499198	821		Control		Uncharacterized protein [Source:UniProtKB/TrEMBL;Acc:F6Y3B5]
ENSXETG00000010848	0.731851196	4716	fam63b	T3-treatment		family with sequence similarity 63, member B [Source:Jamboree;Acc:XB-GENE-990463]
ENSXETG00000011067	0.811321601	1026	irf2bpl	T3-treatment		interferon regulatory factor 2 binding protein-like [Source:Jamboree;Acc:XB-GENE-968642]
ENSXETG00000011167	0.735032657	5536	h2afz	T3-treatment		H2A histone family, member Z [Source:Jamboree;Acc:XB-GENE-485054]
ENSXETG00000011221	0.608700504	3625	eif2s1	T3-treatment		eukaryotic translation initiation factor 2, subunit 1 alpha, 35kDa [Source:Jamboree;Acc:XB-GENE-989630]
ENSXETG00000011674	0.600329882	6151	ociad2	T3-treatment	Non-EP	OCIA domain containing 2 [Source:Jamboree;Acc:XB-GENE-966006]
ENSXETG00000011730	0.650060742	3691		T3-treatment		Uncharacterized protein [Source:UniProtKB/TrEMBL;Acc:F7CRX3]
ENSXETG00000012186	0.749509734	4306	ccnj	T3-treatment	Both EP & Non-EP	cyclin J [Source:Jamboree;Acc:XB-GENE-485597]
ENSXETG00000012208	1.124531048	3486	XB-GENE-5804925	T3-treatment		MGC89226 protein [Source:Jamboree;Acc:XB-GENE-5804925]
ENSXETG00000012223	0.627756418	5946	triap1	T3-treatment		TP53 regulated inhibitor of apoptosis 1 [Source:Jamboree;Acc:XB-GENE-949263]
ENSXETG00000012236	0.600501662	7176	narf	T3-treatment		nuclear prelamin A recognition factor [Source:Jamboree;Acc:XB-GENE-972855]
ENSXETG00000012260	0.583626499	6971	tiparp	T3-treatment		TCDD-inducible poly(ADP-ribose) polymerase [Source:Jamboree;Acc:XB-GENE-1000138]
ENSXETG00000012459	1.013600299	5126	ccnf	Both		cyclin F [Source:Jamboree;Acc:XB-GENE-963729]
ENSXETG00000012575	0.696018317	4306	h3f3b	T3-treatment	EP	H3 histone, family 3B (H3.3B) [Source:Jamboree;Acc:XB-GENE-488758]
ENSXETG00000012766	0.730469393	3691	eif5	Both		eukaryotic translation initiation factor 5 [Source:Jamboree;Acc:XB-GENE-957969]
ENSXETG00000012767	0.704918463	5536	mark3	T3-treatment		MAP/microtubule affinity-regulating kinase 3 [Source:Jamboree;Acc:XB-GENE-867495]
ENSXETG00000012944	0.637362798	7381	foxo6	T3-treatment		forkhead box O6 [Source:Jamboree;Acc:XB-GENE-482993]
ENSXETG00000013007	0.555004017	5536	ugp2	T3-treatment		UDP-glucose pyrophosphorylase 2 [Source:Jamboree;Acc:XB-GENE-974407]
ENSXETG00000013175	0.564245737	1846	gca	T3-treatment	Non-EP	grancalcin, EF-hand calcium binding protein [Source:Jamboree;Acc:XB-GENE-983090]
ENSXETG00000013350	0.85759772	5832	tfg	T3-treatment	EP	trk-fused gene [Source:Jamboree;Acc:XB-GENE-960556]
ENSXETG00000013371	0.631310053	7381	plcb2	T3-treatment		phospholipase C, beta 2 [Source:Jamboree;Acc:XB-GENE-6045997]
ENSXETG00000013492	1.530182345	411	insig1	T3-treatment		insulin induced gene 1 [Source:Jamboree;Acc:XB-GENE-487039]
ENSXETG00000013593	1.041439305	6151	mbd3	Both		methyl-CpG binding domain protein 3 [Source:Jamboree;Acc:XB-GENE-491553]
ENSXETG00000013655	0.650751835	5331	oaz1	T3-treatment		ornithine decarboxylase antizyme 1 [Source:Jamboree;Acc:XB-GENE-490737]
ENSXETG00000013658	0.920856055	4921	dot1l	Both	EP	DOT1-like, histone H3 methyltransferase [Source:Jamboree;Acc:XB-GENE-6033925]
ENSXETG00000013740	0.603216021	616	ca2	T3-treatment	Non-EP	carbonic anhydrase 2 [Source:Jamboree;Acc:XB-GENE-484348]
ENSXETG00000013788	0.553746754	4411	ciapin1	Control	Non-EP	cytokine induced apoptosis inhibitor 1 [Source:Jamboree;Acc:XB-GENE-998244]
ENSXETG00000013975	0.808084657	5126		Control		
ENSXETG00000014257	0.60679815	7791	XB-GENE-5919586	T3-treatment		hypothetical protein MGC75872 [Source:Jamboree;Acc:XB-GENE-5919586]
ENSXETG00000014408	1.811057824	2256	sox4	Both	Both EP & Non-EP	SRY (sex determining region Y)-box 4 [Source:Jamboree;Acc:XB-GENE-480727]
ENSXETG00000014423	0.64384051	6766	stk10	T3-treatment		serine/threonine kinase 10 [Source:Jamboree;Acc:XB-GENE-494456]
ENSXETG00000014519	0.611593039	5536	brd1	Control		bromodomain containing 1 [Source:Jamboree;Acc:XB-GENE-949377]
ENSXETG00000014527	0.550114424	5331	creld2	Control		cysteine-rich with EGF-like domains 2 [Source:Jamboree;Acc:XB-GENE-999014]
ENSXETG00000014529	0.972788949	4716	pim3	Both		pim-3 oncogene [Source:Jamboree;Acc:XB-GENE-922022]
ENSXETG00000014592	1.481857819	5126	runx1t1	T3-treatment		runt-related transcription factor 1; translocated to, 1 (cyclin D-related) [Source:Jamboree;Acc:XB-GENE-481238]
ENSXETG00000014609	0.678706198	5331	decr1	Both		2,4-dienoyl CoA reductase 1, mitochondrial [Source:Jamboree;Acc:XB-GENE-985109]
ENSXETG00000014629	1.3928504	5384	pgpep1	Both		pyroglutamyl-peptidase I [Source:Jamboree;Acc:XB-GENE-961493]
ENSXETG00000014644	0.655770768	5331	sptlc1	T3-treatment		serine palmitoyltransferase, long chain base subunit 1 [Source:Jamboree;Acc:XB-GENE-1004880]
ENSXETG00000014689	0.7224093	6077	tmem67	Control		transmembrane protein 67 [Source:Jamboree;Acc:XB-GENE-985149]
ENSXETG00000014834	0.572675509	411	ptch1	T3-treatment		patched 1 [Source:Jamboree;Acc:XB-GENE-488222]
ENSXETG00000014839	0.799269986	616	cdc14b	T3-treatment		CDC14 cell division cycle 14 homolog B [Source:Jamboree;Acc:XB-GENE-977314]
ENSXETG00000014847	0.613115447	5331	foxo3	T3-treatment		forkhead box O3 [Source:Jamboree;Acc:XB-GENE-945072]
ENSXETG00000014909	0.749529803	1846	arhgap18	T3-treatment	EP	Rho GTPase activating protein 18 [Source:Jamboree;Acc:XB-GENE-988160]
ENSXETG00000014950	1.114596467	411	ccdc136	Both		coiled-coil domain containing 136 [Source:Jamboree;Acc:XB-GENE-5992991]
ENSXETG00000015209	0.772280825	1231	sema4g	T3-treatment		sema domain, immunoglobulin domain (Ig), transmembrane domain (TM) and short cytoplasmic domain, (semaphorin) 4G [Source:Jamboree;Acc:XB-GENE-856594]

ENSXETG00000015656	0.800730025	1231	igfbp1	T3-treatment	EP	insulin-like growth factor binding protein 1 [Source:Jamboree;Acc:XB-GENE-485497]
ENSXETG00000015706	0.588024608	7381	ntm	T3-treatment		neurotrimin [Source:Jamboree;Acc:XB-GENE-6045425]
ENSXETG00000015712	0.889180751	3175	rabl2b	Both	Both EP & Non-EP	RAB, member of RAS oncogene family-like 2B [Source:Jamboree;Acc:XB-GENE-979695]
ENSXETG00000015829	1.28411779	1641		T3-treatment		Uncharacterized protein [Source:UniProtKB/TrEMBL;Acc:F6UG11]
ENSXETG00000015930	1.271505402	1382		Both		
ENSXETG00000015978	0.631119961	7381	ski	T3-treatment	EP	v-ski sarcoma viral oncogene homolog [Source:Jamboree;Acc:XB-GENE-494028]
ENSXETG00000016078	0.653338311	4716	ivd	T3-treatment		isovaleryl-CoA dehydrogenase [Source:Jamboree;Acc:XB-GENE-1008677]
ENSXETG00000016109	0.556918001	3691	gatm	Control		glycine amidinotransferase (L-arginine:glycine amidinotransferase) [Source:Jamboree;Acc:XB-GENE-974514]
ENSXETG00000016147	0.793758254	5946	mtfp1	T3-treatment		mitochondrial fission process 1 [Source:Jamboree;Acc:XB-GENE-1001678]
ENSXETG00000016154	0.689053412	3691	inpp5j	T3-treatment		inositol polyphosphate-5-phosphatase J [Source:Jamboree;Acc:XB-GENE-984978]
ENSXETG00000016479	1.220125515	7421	trpm1	Both		transient receptor potential cation channel, subfamily M, member 1 [Source:Jamboree;Acc:XB-GENE-492528]
ENSXETG00000016685	0.962987792	821	hps5	T3-treatment		Hermansky-Pudlak syndrome 5 [Source:Jamboree;Acc:XB-GENE-5931110]
ENSXETG00000016797	0.866475033	2461		Both		
ENSXETG00000016807	0.797017854	2666	agpat6	T3-treatment		1-acylglycerol-3-phosphate O-acyltransferase 6 (lysophosphatidic acid acyltransferase, zeta) [Source:Jamboree;Acc:XB-GENE-999686]
ENSXETG00000016952	0.644598744	5536	ppm1a	T3-treatment		protein phosphatase, Mg2+/Mn2+ dependent, 1A [Source:Jamboree;Acc:XB-GENE-852654]
ENSXETG00000017065	0.667299542	5741	mhc1a	T3-treatment		major histocompatibility complex class I antigen [Source:Jamboree;Acc:XB-GENE-485307]
ENSXETG00000017179	0.942478568	4175	c18orf45	Both		chromosome 18 open reading frame 45 [Source:Jamboree;Acc:XB-GENE-971853]
ENSXETG00000017326	1.896790901	5741	MGC108117	T3-treatment		MGC108117 protein precursor [Source:RefSeq peptide;Acc:NP_001015694]
ENSXETG00000017395	1.480446922	5536	spag7	Both		sperm associated antigen 7 [Source:Jamboree;Acc:XB-GENE-1010187]
ENSXETG00000017490	0.638835057	411	kiaa0196	T3-treatment		KIAA0196 [Source:Jamboree;Acc:XB-GENE-5781989]
ENSXETG00000017504	0.620592715	5126	nrg1	T3-treatment	Non-EP	neuregulin 1 [Source:Jamboree;Acc:XB-GENE-1011451]
ENSXETG00000017537	0.703813304	5389	c14orf119	T3-treatment		chromosome 14 open reading frame 119 [Source:Jamboree;Acc:XB-GENE-996611]
ENSXETG00000017716	0.927070124	4306	mrps30	Control		mitochondrial ribosomal protein S30 [Source:Jamboree;Acc:XB-GENE-964476]
ENSXETG00000017731	0.644800655	5536	pex11a	T3-treatment		peroxisomal biogenesis factor 11 alpha [Source:Jamboree;Acc:XB-GENE-970930]
ENSXETG00000017801	0.857261504	6356	efnb2	T3-treatment	Both EP & Non-EP	ephrin-B2 [Source:Jamboree;Acc:XB-GENE-488606]
ENSXETG00000017860	1.114659799	5741	mtmr14	T3-treatment		myotubularin related protein 14 [Source:Jamboree;Acc:XB-GENE-5755863]
ENSXETG00000017991	1.893535483	3896		T3-treatment		
ENSXETG00000018210	1.130281728	411	ubxn2a	T3-treatment		UBX domain protein 2A [Source:Jamboree;Acc:XB-GENE-972873]
ENSXETG00000018211	0.627636343	6971	atad2b	T3-treatment		ATPase family, AAA domain containing 2B [Source:Jamboree;Acc:XB-GENE-5956276]
ENSXETG00000018264	0.632917429	5741		Both		
ENSXETG00000018291	0.610825523	5536	cebpa	T3-treatment		CCAAT/enhancer binding protein (C/EBP), alpha [Source:Jamboree;Acc:XB-GENE-853397]
ENSXETG00000018408	0.740624328	5946		T3-treatment		
ENSXETG00000018542	0.565515144	2051		Control		
ENSXETG00000018642	0.694506305	4306	pias3	T3-treatment		protein inhibitor of activated STAT, 3 [Source:Jamboree;Acc:XB-GENE-486632]
ENSXETG00000018646	0.576030792	2871	SH3RF3	Control		SH3 domain containing ring finger 3 [Source:HGNC Symbol;Acc:HGNC:24699]
ENSXETG00000018904	0.59082058	3691	mtrf1	T3-treatment		mitochondrial translational release factor 1 [Source:Jamboree;Acc:XB-GENE-991014]
ENSXETG00000019051	0.85590678	411	klf9	Both	Both EP & Non-EP	Kruppel-like factor 9 [Source:Jamboree;Acc:XB-GENE-483889]
ENSXETG00000019217	0.651820172	5741	pdlim5	T3-treatment	Non-EP	PDZ and LIM domain 5 [Source:Jamboree;Acc:XB-GENE-854114]
ENSXETG00000019242	0.835662324	5536	gmppa	T3-treatment		GDP-mannose pyrophosphorylase A [Source:Jamboree;Acc:XB-GENE-984239]
ENSXETG00000019384	0.529587697	4921	ncbp2	Control		nuclear cap binding protein subunit 2, 20kDa [Source:Jamboree;Acc:XB-GENE-1005786]
ENSXETG00000019410	0.66718963	5946	pafah1b1	T3-treatment		platelet-activating factor acetylhydrolase 1b, regulatory subunit 1 (45kDa) [Source:Jamboree;Acc:XB-GENE-1002843]
ENSXETG00000019456	0.891376579	6151	slc3a2	T3-treatment	EP	solute carrier family 3 (activators of dibasic and neutral amino acid transport), member 2 [Source:Jamboree;Acc:XB-GENE-1009245]
ENSXETG00000019718	0.715213607	6151	cited4	T3-treatment		Cbp/p300-interacting transactivator, with Glu/Asp-rich carboxy-terminal domain, 4 [Source:Jamboree;Acc:XB-GENE-946639]
ENSXETG00000019748	1.086067038	2871	vgll1	T3-treatment		vestigial like 1 [Source:Jamboree;Acc:XB-GENE-982100]
ENSXETG00000020010	0.570222979	3281	dhcr24	T3-treatment		24-dehydrocholesterol reductase [Source:Jamboree;Acc:XB-GENE-979905]
ENSXETG00000020076	0.619781455	3486	tmem130	T3-treatment		transmembrane protein 130 [Source:Jamboree;Acc:XB-GENE-6462848]
ENSXETG00000020416	1.0272873	5331	rxrb	Both		retinoid X receptor, beta [Source:Jamboree;Acc:XB-GENE-480167]
ENSXETG00000020432	0.613240462	2763		T3-treatment		Uncharacterized protein [Source:UniProtKB/TrEMBL;Acc:F7CIZ2]
ENSXETG00000020486	0.637445582	3896	celf4	T3-treatment		CUGBP, Elav-like family member 4 [Source:Jamboree;Acc:XB-GENE-5873699]
ENSXETG00000020685	0.646003054	1436	pcca	T3-treatment	EP	propionyl CoA carboxylase, alpha polypeptide [Source:Jamboree;Acc:XB-GENE-944987]
ENSXETG00000020739	0.609564924	1846	MYO5C	T3-treatment	Non-EP	myosin VC [Source:HGNC Symbol;Acc:HGNC:7604]
ENSXETG00000020930	1.00581806	5536	aldh2	T3-treatment	Non-EP	aldehyde dehydrogenase 2 family (mitochondrial) [Source:Jamboree;Acc:XB-GENE-490836]
ENSXETG00000021046	0.84551864	3281	vmo1	Both		vitelline membrane outer layer 1 homolog (chicken) [Source:Jamboree;Acc:XB-GENE-994821]
ENSXETG00000021098	0.532670829	5331	agxt2l1	Control	Non-EP	alanine-glyoxylate aminotransferase 2-like 1 [Source:Jamboree;Acc:XB-GENE-968831]
ENSXETG00000021543	0.560399198	3477		Both		
ENSXETG00000021645	0.735016425	2461	hal.2	T3-treatment		histidine ammonia-lyase, gene 2 [Source:Jamboree;Acc:XB-GENE-1009089]
ENSXETG00000021760	0.628976111	5536	capza2	T3-treatment		capping protein (actin filament) muscle Z-line, alpha 2 [Source:Jamboree;Acc:XB-GENE-489168]
ENSXETG00000021871	0.575378286	5946	dusp6	T3-treatment		dual specificity phosphatase 6 [Source:Jamboree;Acc:XB-GENE-978216]
ENSXETG00000022090	0.679071809	821		Both		
ENSXETG00000022091	1.008327759	3795		Both		
ENSXETG00000022103	1.295138632	6151		Both		
ENSXETG00000022107	1.137341698	4716		Both		
ENSXETG00000022222	0.636533218	4921	etfb	T3-treatment		electron-transfer-flavoprotein, beta polypeptide [Source:Jamboree;Acc:XB-GENE-956098]
ENSXETG00000022360	1.091474998	4101	slc2a11	Both		solute carrier family 2 (facilitated glucose transporter), member 11 [Source:Jamboree;Acc:XB-GENE-980809]
ENSXETG00000022389	0.656413192	5331	slc25a1	T3-treatment	EP	solute carrier family 25 (mitochondrial carrier; citrate transporter), member 1 [Source:Jamboree;Acc:XB-GENE-491686]
ENSXETG00000022397	0.782153419	4921		T3-treatment		
ENSXETG00000022525	0.607636699	3076	dll1	T3-treatment		delta-like 1 [Source:Jamboree;Acc:XB-GENE-479210]
ENSXETG00000022643	1.328316594	4716	c1orf55	T3-treatment		chromosome 1 open reading frame 55 [Source:Jamboree;Acc:XB-GENE-5807809]
ENSXETG00000022909	0.827619088	1641	kiaa0232	T3-treatment		KIAA0232 [Source:Jamboree;Acc:XB-GENE-5888041]
ENSXETG00000023023	0.793135237	616		Both		
ENSXETG00000023061	0.646104839	616	xrcc3	T3-treatment		X-ray repair complementing defective repair in Chinese hamster cells 3 [Source:Jamboree;Acc:XB-GENE-1016741]
ENSXETG00000023106	0.634546958	6356	pmpcb	T3-treatment		peptidase (mitochondrial processing) beta [Source:Jamboree;Acc:XB-GENE-999999]
ENSXETG00000023129	0.886038003	5126	ier5	Both	EP	immediate early response 5 [Source:Jamboree;Acc:XB-GENE-1004819]
ENSXETG00000023202	0.800996233	1641	uba52	T3-treatment		ubiquitin A-52 residue ribosomal protein fusion product 1 [Source:Jamboree;Acc:XB-GENE-939925]

ENSXETG00000023220	0.6116615	5126		T3-treatment		uncharacterized protein LOC100145115 [Source:RefSeq peptide;Acc:NP_001120099]
ENSXETG00000023263	1.881293469	7791	cops5	T3-treatment		COP9 constitutive photomorphogenic homolog subunit 5 [Source:Jamboree;Acc:XB-GENE-952827]
ENSXETG00000023332	0.530257177	3076	galnt2	Control		UDP-N-acetyl-alpha-D-galactosamine:polypeptide N-acetylgalactosaminyltransferase 2 (GalNAc-T2) [Source:Jamboree;Acc:XB-GENE-980077]
ENSXETG00000023408	0.573849381	5741	sdha	T3-treatment		succinate dehydrogenase complex, subunit A, flavoprotein (Fp) [Source:Jamboree;Acc:XB-GENE-956942]
ENSXETG00000023564	0.720251162	3896	cndp1	T3-treatment		carnosine dipeptidase 1 (metallopeptidase M20 family) [Source:Jamboree;Acc:XB-GENE-969925]
ENSXETG00000023610	0.636469317	2256	ghsr	T3-treatment		growth hormone secretagogue receptor [Source:Jamboree;Acc:XB-GENE-484475]
ENSXETG00000023684	0.727461219	6766	znf827	T3-treatment		zinc finger protein 827 [Source:Jamboree;Acc:XB-GENE-1033048]
ENSXETG00000024399	0.900943407	7176	THRA	T3-treatment		thyroid hormone receptor, alpha [Source:RefSeq peptide;Acc:NP_001039261]
ENSXETG00000024449	0.921639937	4558	ppp1r11	T3-treatment		protein phosphatase 1, regulatory (inhibitor) subunit 11 [Source:Jamboree;Acc:XB-GENE-999517]
ENSXETG00000024480	0.760240323	5946	ppp1cc	T3-treatment		protein phosphatase 1, catalytic subunit, gamma isozyme [Source:Jamboree;Acc:XB-GENE-967934]
ENSXETG00000024496	1.026685959	7791	XB-GENE-5810927	T3-treatment		hypothetical protein LOC100036876 [Source:Jamboree;Acc:XB-GENE-5810927]
ENSXETG00000024509	1.437572526	1231	slc25a23	Both		solute carrier family 25 (mitochondrial carrier; phosphate carrier), member 23 [Source:Jamboree;Acc:XB-GENE-5998763]
ENSXETG00000024677	0.590187609	5331	ndufa3	Both		NADH dehydrogenase (ubiquinone) 1 alpha subcomplex, 3, 9kDa [Source:Jamboree;Acc:XB-GENE-1006323]
ENSXETG00000024680	0.599198102	616		T3-treatment		
ENSXETG00000024770	0.556932815	5331		Control		Uncharacterized protein [Source:UniProtKB/TrEMBL;Acc:F7BMN9]
ENSXETG00000024837	0.605949449	493		T3-treatment		
ENSXETG00000024875	1.05854878	2666	c7orf11	Both		chromosome 7 open reading frame 11 [Source:Jamboree;Acc:XB-GENE-942374]
ENSXETG00000024911	0.943846094	7586	tgfa	T3-treatment		transforming growth factor, alpha [Source:Jamboree;Acc:XB-GENE-852694]
ENSXETG00000024974	0.957426537	1210		T3-treatment		
ENSXETG00000024985	0.715945289	5741	c19orf63	T3-treatment		chromosome 19 open reading frame 63 [Source:Jamboree;Acc:XB-GENE-5788294]
ENSXETG00000024993	0.678463782	4101		Both		
ENSXETG00000024995	0.741665796	7060		Both		
ENSXETG00000024996	0.945756821	1641		Both		
ENSXETG00000025000	0.636735799	654		Both		
ENSXETG00000025001	0.899952944	5856		Both		
ENSXETG00000025007	0.718542023	5331	n4bp1	T3-treatment		nedd4 binding protein 1 [Source:Jamboree;Acc:XB-GENE-1216138]
ENSXETG00000025094	0.571774656	4101		T3-treatment		
ENSXETG00000025097	0.820160785	6971	hmgn1	T3-treatment		high mobility group nucleosome binding domain 1 [Source:Jamboree;Acc:XB-GENE-5782073]
ENSXETG00000025179	0.630925351	821		Control		
ENSXETG00000025214	0.698840157	411	bnip3	T3-treatment	Both EP & Non-EP	BCL2/adenovirus E1B 19kDa interacting protein 3 [Source:Jamboree;Acc:XB-GENE-971175]
ENSXETG00000025990	1.034717537	5050	brp44l	Both		brain protein 44-like [Source:Jamboree;Acc:XB-GENE-970069]
ENSXETG00000026175	0.59423579	5536	timm23	T3-treatment		translocase of inner mitochondrial membrane 23 homolog [Source:Jamboree;Acc:XB-GENE-994532]
ENSXETG00000026398	0.675752127	2256		T3-treatment		
ENSXETG00000026537	1.028306728	3486	gadd45g	Both		growth arrest and DNA-damage-inducible, gamma [Source:Jamboree;Acc:XB-GENE-481955]
ENSXETG00000026573	0.653136807	2461		Both		
ENSXETG00000026585	0.673202298	5331	nudt4	T3-treatment		nudix (nucleoside diphosphate linked moiety X)-type motif 4 [Source:Jamboree;Acc:XB-GENE-943736]
ENSXETG00000026613	0.551876403	411		T3-treatment		
ENSXETG00000027016	0.591082662	4410		T3-treatment		
ENSXETG00000027093	0.920425549	6356		T3-treatment		uncharacterized protein LOC100170590 [Source:RefSeq peptide;Acc:NP_001123834]
ENSXETG00000027097	0.684404515	4921	arpp19	T3-treatment		cAMP-regulated phosphoprotein, 19kDa [Source:Jamboree;Acc:XB-GENE-953906]
ENSXETG00000027128	0.563404735	1231	uqcrq	T3-treatment		ubiquinol-cytochrome c reductase, complex III subunit VII, 9.5kDa [Source:Jamboree;Acc:XB-GENE-977452]
ENSXETG00000027133	0.667628105	616		T3-treatment		
ENSXETG00000027149	0.627618807	411		T3-treatment		Uncharacterized protein [Source:UniProtKB/TrEMBL;Acc:F7E699]
ENSXETG00000027207	0.730305533	5126	ufm1	T3-treatment		ubiquitin-fold modifier 1 [Source:Jamboree;Acc:XB-GENE-973175]
ENSXETG00000027282	0.77005625	1436	flvcr2	T3-treatment		feline leukemia virus subgroup C cellular receptor family, member 2 [Source:Jamboree;Acc:XB-GENE-1016322]
ENSXETG00000027459	0.694000653	6151	c7orf23	Both		chromosome 7 open reading frame 23 [Source:Jamboree;Acc:XB-GENE-952756]
ENSXETG00000027482	0.831291034	1026	fbxo6	T3-treatment		F-box protein 6 [Source:Jamboree;Acc:XB-GENE-5797733]
ENSXETG00000027534	0.624596935	4101	hoxa4	T3-treatment		homeobox A4 [Source:Jamboree;Acc:XB-GENE-486676]
ENSXETG00000027622	0.844216923	3896	idh2	T3-treatment		isocitrate dehydrogenase 2 (NADP+), mitochondrial [Source:Jamboree;Acc:XB-GENE-976315]
ENSXETG00000027641	0.573501577	5331	actg1	T3-treatment		actin, gamma 1 [Source:Jamboree;Acc:XB-GENE-491618]
ENSXETG00000027647	1.015082736	3076		T3-treatment		
ENSXETG00000027849	0.606264265	581	HIST2H2AB	T3-treatment		histone cluster 1, H2aj [Source:RefSeq peptide;Acc:NP_001004821]
ENSXETG00000027887	1.079704572	5741	trib2	T3-treatment		tribbles homolog 2 [Source:Jamboree;Acc:XB-GENE-489426]
ENSXETG00000028367	0.759261007	6633		Control		
ENSXETG00000028378	0.713175656	4170		T3-treatment		
ENSXETG00000028734	0.635904988	2382	U2	Control		U2 spliceosomal RNA [Source:RFAM;Acc:RF00004]
ENSXETG00000028912	0.709631379	3924		Control		
ENSXETG00000028917	0.602152831	206	xtr-mir-200a	T3-treatment		xtr-mir-200a [Source:miRBase;Acc:MI0004945]
ENSXETG00000029004	0.693566044	3508	xtr-mir-19b-1	T3-treatment		xtr-mir-19b-1 [Source:miRBase;Acc:MI0004960]
ENSXETG00000029132	1.039972972	616	xtr-mir-181a-2	T3-treatment		xtr-mir-181a-2 [Source:miRBase;Acc:MI0004866]

*Based the published information by Sun et al. (2013), Expression profiling of intestinal tissues implicates novel genes and pathways essential for adult stem cell development. Endocrinology 154 (11), 4396–4407. Note that the regulation of most of the genes are unknow as they were not found on the microarray used by Sun et al. (2013), likely due to the use of different gene names on the microarry vs. genomic chips. EP: epithelium; non-EP: the rest of the intestine.
